# Advancing Cancer-Targeted Nanotherapies with Tumor Homing Peptides

**DOI:** 10.1021/acsptsci.5c00241

**Published:** 2025-06-11

**Authors:** Antonella Rocchi, Tambet Teesalu, Christian Celia

**Affiliations:** † Department of Pharmacy, 60220 University of Chieti − Pescara “G. d’Annunzio”, Via dei Vestini 31, Chieti 66100, Italy; ‡ Laboratory of Precision and Nanomedicine, Institute of Biomedicine and Translational Medicine, University of Tartu, Ravila 14b, Tartu 50411, Estonia; § Materials Research Laboratory, University of California, Santa Barbara, California 93106, United States; ∥ Institute of Nanochemistry and Nanobiology, School of Environmental and Chemical Engineering, Shanghai University, Shanghai 200444, China; ⊥ SCM Nutraceutici Universitari Srl, Strada degli Oliveti 73, Chieti 66100, Italy

**Keywords:** nanoparticle, precision medicine, active targeting, homing peptide, iRGD, C-end Rule peptides

## Abstract

The development of smart nanoparticles for precision medicine in anticancer drug delivery and controlled release has the potential to revolutionize cancer treatment. This progress is driven by expanding body of preclinical and early clinical studies on anticancer nanoparticles. Despite these advancements, a critical challenge remains achieving a selective and efficient tumor accumulation, along with effective extravasation and deep tissue penetration of nanoparticles into the tumor parenchyma. Affinity targeting strategies using ligands such as homing peptides and antibodies leverage molecular recognition to enhance selectivity, efficacy, and therapeutic activity by directing them to tumor-specific receptors or biomarkers, thereby minimizing off-target effects and improving drug delivery efficiency. Tumor-homing C-end Rule (CendR) peptides, such as prototypic iRGD peptide, represent a promising strategy due to their unique multistep mechanism that facilitates both selective targeting and enhanced tissue penetration. This review highlights key milestones in the evolution of peptide-targeted nanoparticles, underscoring their role in advancing precision nanomedicine.

## Graphical abstract

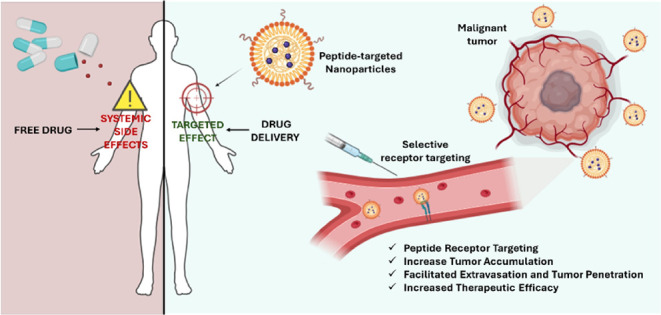

## 1 Current Therapeutic Approach to Cancer Management

Cancer remains one of the leading causes of death worldwide, second only to cardiovascular diseases.^1^ While advancements in early diagnosis and development of molecular targeted therapies have improved long-term survival rates for many cancer types,^2^ cancer remains a major global health challenge, with ∼ 19.3 million new cancer cases and ∼ 10 million deaths reported in 2020 alone.^3^ Current therapeutic protocols, tailored to the specific cancer type, generally involve the combined or individual use of surgery, radiotherapy, and chemotherapy.^4^ Surgery remains essential for managing most localized solid tumors, whereas systemic treatments are used for the prevention or treatment of distant metastases.^5^ Radiotherapy is used in ∼ 50% of patients at various stages of care,^6^ delivering high-energy radiation to damage the DNA of cancer cells, thereby inhibiting their capacity to proliferate.^7^ Advancements in radiation therapy include introduction of techniques such as proton beam therapy and the carbon ion therapy allow for more precise targeting of tumors, reducing damage to surrounding healthy tissues and minimizing side effects.^8^

Systemic strategies such as small-molecule targeted therapies and antibody-based immunotherapies have significantly improved the personalized treatment, precision medicine and patient outcomes.^9^ Nevertheless, conventional cytotoxic agents have a main role in anticancer therapy and in many therapeutic regimens, and even next-generation chemotherapeutics, despite delivering marked survival benefits and, in some cases, curative results, had a dose – dependent toxicities.^10–12^

Consequently, patients commonly experience adverse effects that impair quality of life and may lead to dose restriction and even to treatment discontinuation. Common side effects include fatigue, nausea, vomiting, hair loss, loss of appetite, taste alterations, and gastrointestinal disturbances. More serious side effects may include severe bone marrow suppression leading to anemia, leukopenia, or thrombocytopenia; increased risk of infections; organ toxicity affecting the liver, kidneys, or heart; severe gastrointestinal complications such as mucositis, ulcers, or bleeding; and neurological effects such as peripheral neuropathy, cognitive impairment, or seizures (Table 1).^13^

**Table 1:** Commonly Used Chemotherapeutic Agents and Their Systemic Side Effects

Class of chemotherapeutic agents	Examples	Mechanism of action	Systemic side effects	References
Alkylating agents	Cyclophosphamide, Temozolomide	Direct DNA damage, inhibits cancer cell replication	Myelosuppression, nephrotoxicity, gastrointestinal disturbances, potential neurotoxicity (e.g., with Temozolomide)	^14^
Antimetabolites	Methotrexate, 5-Fluorouracil, Gemcitabine	Inhibit DNA/RNA synthesis, preventing cell proliferation	Mucositis, immunosuppression, hepatotoxicity, pulmonary toxicity (e.g., with gemcitabine)	^15^
Anthracyclines	Doxorubicin, Epirobicine, Daunorubicin	DNA intercalation, free radical generation	Dose-dependent cardiotoxicity (heart failure risk), myelosuppression, alopecia	^16^
Plant Alkaloids	Vincristine, Etoposide, Vinblastine	Disrupt microtubule formation or DNA repair processes	Neurotoxicity (peripheral neuropathy), bone marrow suppression, gastrointestinal toxicity	^17^
Taxanes	Paclitaxel, Docetaxel, Cabazitaxel	Stabilize microtubules, causing cell cycle arrest	Myelosuppression, hypersensitivity reactions, peripheral neuropathy	^18^
Topoisomerase Inhibitors	Irinotecan, Topotecan	Inhibit topoisomerase enzymes, prevent DNA repair	Severe diarrhea (e.g., irinotecan), myelosuppression, alopecia	^19^
Platinum Compound	Carboplatin, Oxaliplatin, Cisplatin	Form DNA cross-linkers, inhibit DNA replication and repair	Nephrotoxicity, neurotoxicity, ototoxicity (hearing loss), myelosuppression	^20^

Although conventional chemotherapies can be injected at their maximum tolerated dose (MTD), with to dose-limiting toxicities, small-molecule targeted agents and monoclonal antibodies are typically dosed based on their pharmacodynamic end points rather than MTD. This dosing paradigm significantly decreased the incidence of conventional cytotoxic side effects.^21^ Nevertheless, these “precision” therapies have some drawbacks like on-target toxicities (e.g., rash with EGFR inhibitors),^22^ hypertension with VEGF blockers^23^ and off-target effects (e.g., cardiotoxicity, hepatotoxicity) that can still limit their clinical use.^24^ Moreover, heterogeneous target expression, poor tissue penetration, and acquired drug resistance often need some combination regimens that may reintroduce the systemic toxicity.^25^

These persistent challenges have spurred interest in precision nanomedicine, particularly tumor-homing peptide-functionalized nanoparticles, which combine ligand-mediated targeting with engineered delivery nanoplatforms.^26^ By enhancing the tumor accumulation and reducing the systemic exposure, such biomaterials aim to preserve the advantages of targeted dosing while mitigating both classic and mechanism-specific toxicities.^27^

### 1.1 Antibody-Based Therapeutics and Conjugates

Monoclonal antibodies (mAbs) have revolutionized cancer therapy by their selective specificity and potent antitumor activity.^28^ Engineered from a single hybridoma or recombinant cell line,^29^ mAbs selectively bind tumor-associated antigens, such as EGFR (e.g., cetuximab for colorectal and head and neck cancers,^30^ VEGFA (bevacizumab for metastatic colorectal cancer,^31^ HER2 (trastuzumab for HER2-positive breast and gastric cancers,^32^ CD20 (rituximab for non-Hodgkin lymphomas,^33^ and PD-L1 (atezolizumab for nonsmall cell lung cancer and urothelial carcinoma,^34^ thereby disrupting critical signaling pathways, blocking receptor–ligand interactions, and recruiting immune effector functions (ADCC, CDC, checkpoint inhibition).^35^

Bispecific antibodies (bsAbs) have emerged as a next-generation product capable of simultaneously bind two distinct targets, typically a tumor antigen and an immune effector molecule, thus enhancing tumor specificity and immune-mediated cytotoxicity.^36^ Clinically approved bsAbs include amivantamab (EGFR/MET, NSCLC),^37^ blinatumomab (CD3/CD19, B-ALL),^38^ and epcoritamab (CD3/CD20, DLBCL).^39^

Antibody-drug conjugates (ADCs) had a precise and personalized targeting with the potency of cytotoxins via cleavable linkers.^40^ Notable FDA-approved ADCs include trastuzumab-emtansine (TDM1) for HER2-positive breast cancer^41^ and brentuximab-vedotin for CD30-positive lymphomas.^42^ By releasing their payload at the tumor site, ADCs have specificity limitations, though their efficacy in solid tumors remains constrained by heterogeneous antigen expression and limited tissue penetration.^43^

Despite these advances, mAbs, bsAbs, and ADCs have key challenges in solid malignancies, like tumor heterogeneity, poor penetration into dense tumor stroma, and off-tumor toxicity, which limit their therapeutic efficacy and wide application.^44,45^ Innovative drug delivery strategies and rational molecular design have a significant impact for therapeutic effectiveness and clinical translation of nanoparticles. In particular, combination therapy with antibodies, chemotherapeutic drugs, immune checkpoint blockade, and nanocarriers have a significant impact and therapeutic efficacious in preclinical and clinical studies and provide a potent and precise effectiveness for the treatment of solid tumors, their mass reduction and inhibition of tumor growing.^46,47^

## 2 Drug Delivery Nanosystems: The Revolution of Controlled Release

Paul Ehrlich postulated that an ideal therapeutic agent would be one capable of selectivity reaching its target, taking to a “magic bullet” (*magische kugel*).^48^

Drug delivery systems based on nanoparticles, represent the closest realization of Ehrlich’s vision. These systems mark a significant advancement toward a more selective and safer therapeutic approach, particularly in oncology.^49,50^ Due to their unique physicochemical properties and compositions, nanoparticles can encapsulate drugs and enable selective targeting of tumor tissue.^51^ Nanoformulation significantly influences the pharmacokinetics of small-molecule drugs by reducing metabolism and premature clearance, thereby extending blood circulation time and enhancing drug accumulation at the tumor site.^52^ Nanoparticles used in biomedical applications can be classified into organic, inorganic, and biological categories, each with distinct properties and functions (Figure 1).

**Figure 1:**
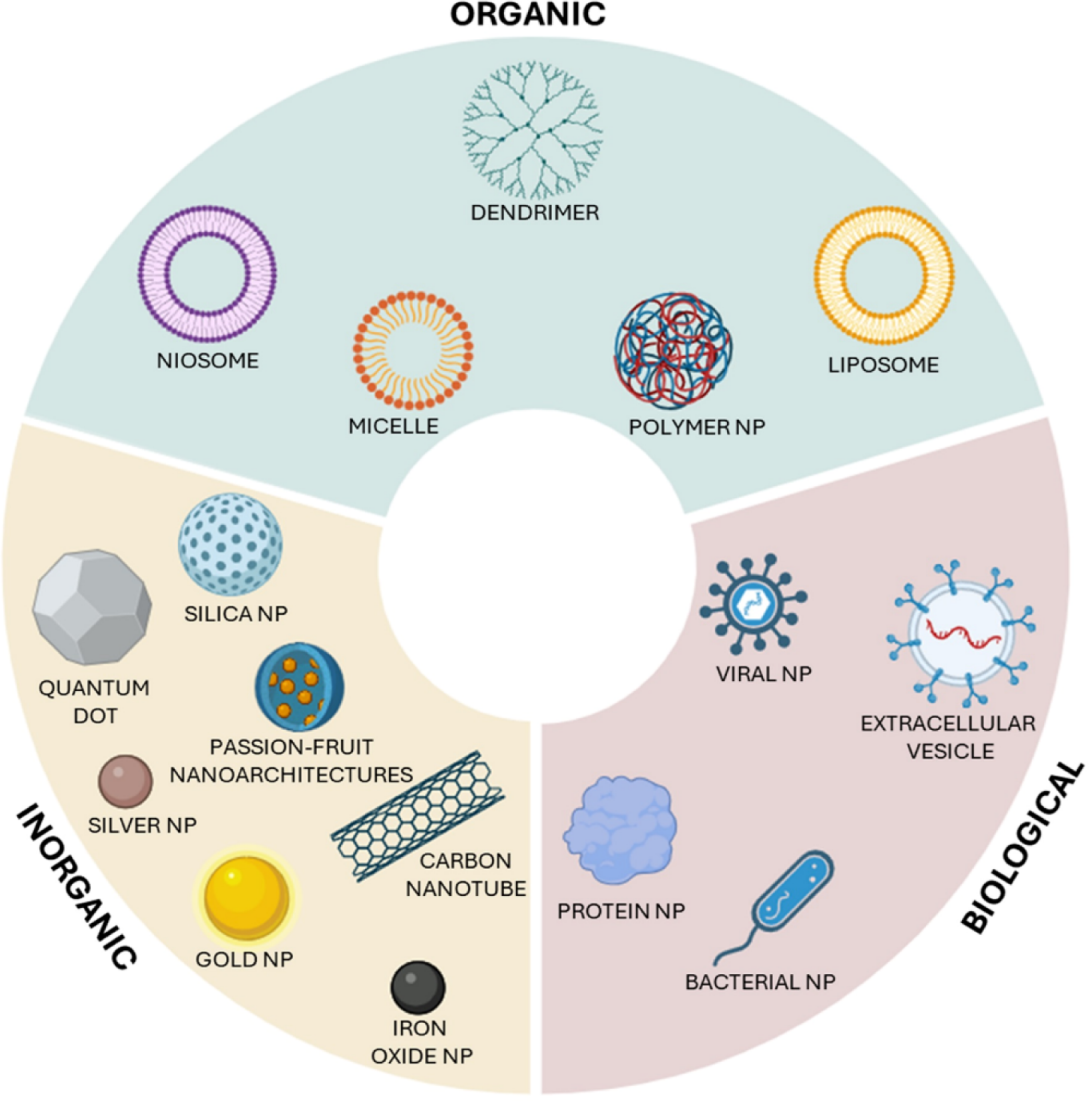
Main classes of nanoparticles developed for biomedical applications. The organic class includes micelles, dendrimers, liposomes, niosomes, and polymeric nanoparticles, which are primarily designed for drug delivery due to their biocompatibility, tunable surface properties, and versatile drug-loading capabilities. The inorganic class comprises quantum dots, gold nanoparticles, iron oxide nanoparticles, carbon nanotubes, silver nanoparticles, silica nanoparticles and “passion-fruit” nanoarchitectures, characterized by their unique physical and chemical properties that make them valuable for imaging, therapy, and diagnostics. The biological class consists of viral nanoparticles, protein-based nanoparticles, extracellular vesicles (EVs), and bacteria-derived nanoparticles, leveraging natural biological systems to enhance targeting specificity and reduce immunogenicity, making them promising candidates for advanced therapeutic and diagnostic applications. Adapted from.^53^ *Created with BioRender.com*.

The evolution of nanoparticles has been rapid, with dozens of formulations developed and optimized to expand clinical applications, improve selectivity and efficacy, and reduce treatment toxicity, establishing a presence in areas beyond oncology.^54^ The growing interest in nanomedicine is reflected in the increasing volume of research, with ∼ 100,000 studies published between January 1999 and December 2022, 80% of which appeared between 2015 and 2023.^55^ Liposomes have become the most studied and tested nanoparticles for therapeutic applications, paving the way for more targeted and safer therapies.^56,57^ Their discovery dates to the 1960s and is credited to British hematologist Alec Douglas Bangham. While studying the properties of phospholipids at the Babraham Institute in Cambridge, Bangham and his colleague R.W. Horne observed that when suspended in water, phospholipids spontaneously formed closed vesicles, structurally like cellular membranes, comprising one or more lipid bilayers. These structures, initially referred to as “Banghasomes” or “multilamellar smectic mesophases”, were later renamed “liposomes” by American physician Gerald Weissmann.^58^ Weissmann suggested this more user-friendly term, drawing a parallel to “lysosome,” a cellular organelle. Bangham further demonstrated that the lipid bilayer encapsulated an aqueous core capable of containing hydrophilic molecules, effectively shielding them from the external environment. In 1965, Bangham, together with Jeff Watkins and Standish, published a paper describing these structures, inadvertently laying the foundation for the liposome industry.^59^

While liposomes pioneered the field and remain one of the most clinically established platforms, other classes of nanoparticles have gained traction, offering distinct advantages in addressing specific medical challenges.

Polymeric nanoparticles, including micelles and dendrimers, are engineered for controlled drug release and improved solubility of poorly water-soluble drugs, making them valuable for managing chronic diseases and neurological disorders.^60^ Inorganic nanoparticles, such as gold and iron oxide nanoparticles, have revolutionized imaging, diagnostics, and theranostics, by enabling tracking of therapeutic responses while simultaneously delivering treatment.^61^ Quantum dots and silica nanoparticles further enhance these capabilities though their highly tunable optical properties, improving precision in biomedical imaging and biosensing.^62^ Additionally, biological nanoparticles, including EVs and virus-like particles, leverage natural biomimicry to enhance biocompatibility and targeting while minimizing immune system activation. These systems hold great promise in regenerative medicine, gene therapy, and immunotherapy, and demonstrate potential for management of neurodegenerative diseases, infectious diseases, and inflammatory conditions.^63,64^

Nanoparticles are versatile and have different properties and compositions. Biomaterials such as lipids, polymers, surfactants, and their combinations as well as hybrid materials composed of natural and synthetic components, are commonly used to engineer nanoparticles with tailored surface properties for targeted therapeutic and/or diagnostic payload delivery. Combination of biomaterials and their relative advantages are suitable to have different types of nanoparticles for controlled drug release and precision medicine as well as active and passive targeting^56^ (Figure 2).

**Figure 2:**
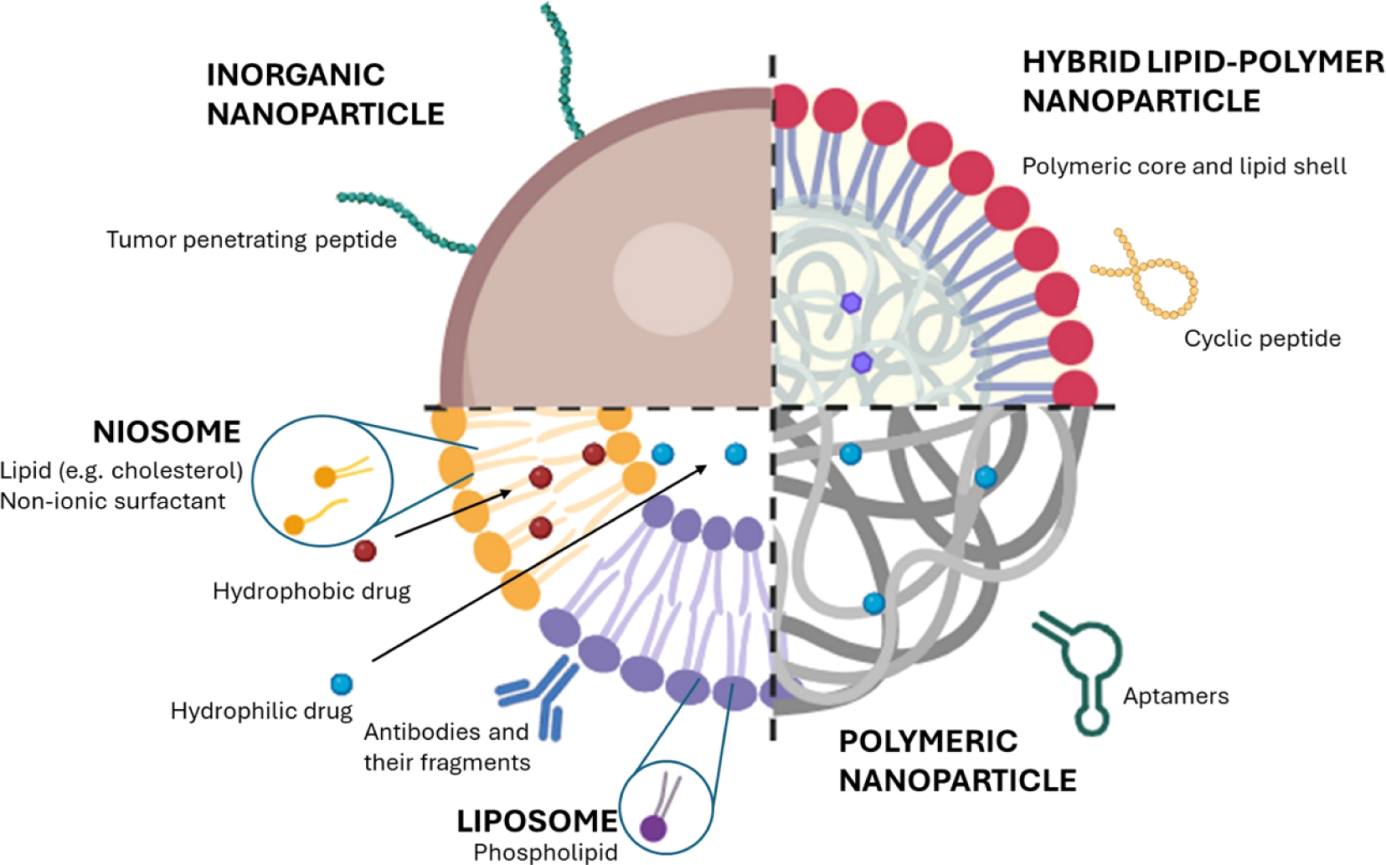
Schematic representation of the main classes of therapeutic nanoparticles for active and passive targeting. Adapted from^65^ *Created with BioRender.com*.

Doxil, the first FDA-approved liposomal formulation of doxorubicin, remains a blockbuster in nanomedicine.^66^ However, its clinical development had some critical issues in the manufacture process. In 2011, Doxil was temporarily supply suspension in the pharmaceutical market due to a production-site quality failures, which led to inconsistent control of nanoparticle size, drug load, and colloidal stability at large scale production. To address the shortage, the FDA temporarily authorized the replacement with Lipodox (Sun Pharma) in early 2012. This incident demonstrated the difficulty of maintaining batch-to-batch consistency in critical parameters (nanoparticle size, drug loading, and colloidal stability) during the scale-up from bench laboratory, with small amount of drug, to commercial production, with large amount of drug.^67^ Nevertheless, in recent years, microfluidic-assisted synthesis has emerged as a promising solution, offering continuous, automated control over mixing, reaction time, and temperature.^68^ By running multiple microreactors in parallel under precisely defined flow conditions, this approach can yield uniform nanoparticles and consistent encapsulation efficiencies across extended production runs.^69^

The approved therapeutic anticancer nanodrugs, along with their key characteristics, are summarized in Table 2 (Adapted from.^70^

**Table 2:** FDA/EMA Classification of Nanoparticles Approved for Anticancer Therapy^a^

Nanostructure	Name	Description	Therapeutic indication	Approval
Lipid-based nanoparticles	Doxil/Caelyx	PEGylated (polyethylene glycol) Doxorubicin- liposome	Kaposi’s sarcoma, ovarian cancer, multiple myeloma	FDA (1995) EMA (1996)
	DaunoXome	Non-PEGylated daunorubicin liposome	Kaposi’s sarcoma	FDA (1996)
	Myocet	Non-PEGylated Doxorubicin- liposome	Metastatic breast cancer	EMA (2000)
	Mepact	Non-PEGylated-mifamurtide liposome	Osteosarcoma	EMA (2009)
	Marqibo	Non-PEGylated vincristine liposome	Acute lymphoid leukemia	FDA (2012)
	Onivyde	PEGylated irinotecan liposome	Pancreatic cancer, Colorectal cancer	FDA (2015)
	Vyxeos	Liposomal formulation of cytarbicine and daunorubicin	Acute myeloid leukemia	EMA (2018)
Gel embedding nanoparticles	Ameluz	5-aminolevulinic acid	Superficial and/or nodular basal cell carcinoma	EMA (2011)
Protein-drug conjugated	Oncaspar	Covalent conjugate of l-asparaginase with mPEG, MSP, Heptahydrate, and NaCl	Acute lymphoblastic leukemia	FDA (1994)
	Ontak	Recombinant cytotoxic protein composed of diphtheria toxin fragments A and B (Met1-Thr387)-His and human IL-2 (Ala1-Thr133)	Cutaneous T-cell lymphoma	FDA (1999)
	Eligard	Polymeric matrix of leuprorelin acetate composed of PLGA (85:15) NMP and LA	Prostate cancer	FDA (2002)
	Abraxane	Colloidal suspension without solvent of paclitaxel bound to albumin (active substance) in the form of a spherical nanoparticle	Breast cancer Nonsmall lung cancer Pancreatic cancer	FDA (2005)
	Kadcyla	Trastuzumab, covalently linked to DM1 via the stable thioether linker MCC	HER2^+^ breast cancer	EMA (2013) FDA (2013)
	Pazenir	Paclitaxel formulated as albumin bound nanoparticles. Powder for dispersion for infusion	Metastatic breast cancer, metastatic adenocarcinoma of the pancreas, nonsmall cell lung cancer	EMA (2019)
Metallic nanoparticles	NanoTherm	Nanoparticles of superparamagnetic iron oxide coated with amino silane	Glioblastoma, Prostate cancer, Pancreatic cancer	EMA (2013)

a MSP = Monosodium Phosphate; PLGA = Poly­(lactic-*co*-glycolic acid); NMP = *N*-methyl-2-pyrrolidone; MCC = Maleimidomethyl cyclohexane-1-carboxylate; LA = Leuprorelin Acetate.

In addition, the unique physicochemical properties of nanoparticles can be exploited for multimodal applications in cancer theranostics, an emerging field that integrates therapeutic and diagnostic functions within a single platform.^71^ Theranostic nanoparticles enable real-time monitoring of drug distribution, tumor targeting, and treatment response while simultaneously delivering therapeutic agents, thereby facilitating personalized medicine approaches. Currently, 15 nanoparticle-based therapeutic and imaging agents have been approved by the FDA and EMA for treatment of malignant diseases.^70^

The use of nanoparticles as drug delivery systems offers the promise of enhanced pharmacokinetics, increased biodistribution, and reduced systemic toxicity compared to conventional chemotherapy. While many of the chemotherapeutic payloads encapsulated in nanoparticles have already been approved in their free forms, the clinical outcomes of nanoparticle-based formulations have yet to show a significant improvement in overall survival.^50,72^

The promise of localized concentration and reduced systemic exposure provided by these “magic bullets” has been met with significant challenges, particularly in achieving selective accumulation at the tumor site.^73^ Despite advancements in nanoparticle design strategies, the rapid clearance of nanoparticles, difficulties in penetrating biological barriers, such as endothelial lining of blood vessels, and the heterogeneity of tumor microenvironment, remain major obstacles.

Tumor tropism of currently approved anticancer nanomedicines relies on the Enhanced Permeability and Retention (EPR) effect, based on the premise that tumor blood vessels, due to increased angiogenesis, elevated production of permeability mediators (e.g., like prostaglandins, bradykinin, nitric oxide, and vascular endothelial growth factor) endothelial discontinuity and other structural abnormalities, have higher permeability than normal vasculature, facilitating nanoparticle accumulation in the tumor.^74^ However, these properties are uneven across different tumor regions. Furthermore, the EPR effect is counterbalanced by the high interstitial pressure commonly seen in solid tumors, caused by inadequate drainage from dysfunctional lymphatic vessels.^75–77^

In terms of tumor perfusion, four main regions are identified: a deep avascular necrotic region, a seminecrotic zone, an area with microcirculation, and an advancing vascularized front in the peripheral zone.^78^ Nanoparticles must navigate through these regions, with poor perfusion in the avascular and seminecrotic areas limiting the delivery of therapeutic agents. Imaging studies have shown that doxorubicin, for example, penetrates only 40–50 μm (approximately 3–5 cell diameters) into the tumor mass, leaving pockets of tumor cells exposed to minimal drug doses.^79^ This complexity highlights the challenges in achieving uniform systemic delivery, extravasation, and penetration of nanoparticles within the solid tumors.^80^

## 3 Toward Precision Nanomedicine: Vascular ZIP Codes and Tumor Homing Peptides

Affinity targeting of molecular markers overexpressed in diseased tissue can be used to achieve localized drug accumulation, effectively mimicking a topical concentration while minimizing systemic distribution and reducing associated side effects.^81^

The concept of molecular “zip codes” was inspired by observations of embryonic development, where cells are involved in multiple recognition events to migrate and organize into defined structures.^82,83^ These phenomena led to the hypothesis that specific adhesion molecules might function as molecular “postal codes,” mediating precise cell–cell and cell-tissue recognition.^84^ In systemic drug delivery, the vascular barrier serves as the primary gateway to tissues and disease sites. At the molecular level, endothelial cells express tissue- and condition-specific markers, referred to as “vascular ZIP codes”, which are accessible throughout the body and can guide targeted therapeutic approaches. This concept was advanced in cancer biology by Erkki Ruoslahti, who hypothesized that a defective molecular signature on the surface of metastatic cancer cells might explain their ability to spread throughout the body.^85^

A major breakthrough in characterization of vascular ZIP codes and developing target specific homing peptides occurred in 1996 when Erkki Ruoslahti’s laboratory pioneered the use of *in vivo* phage display to discover tissue- and disease-specific vascular homing peptides.^86^ In this technique, peptide libraries are genetically engineered onto the surface proteins of bacteriophages, creating a direct physical link between the displayed peptide (phenotype) and its encoding DNA (genotype). The library is intravenously injected into live animals, allowing the phages to circulate and interact with the endothelium of different tissues. After systemic circulation, target organs are harvested, and bound phages are recovered. The procedure integrates positive selection in the tissue of interest with negative selection against off-target organs to enhance specificity. Selected phages are then reinjected in subsequent rounds of biopanning, enriching for clones with high affinity toward the target vasculature^87,88^ (Figure 3). This approach has led to the discovery of multiple peptides with unique and highly selective homing properties and allowed development of maps of differentially expressed vascular receptors.^89,90^ Being an agnostic technique, phage display has led to the identification of a multitude of peptides that rely on different recruitment receptors and downstream interaction pathways. While all tumor-homing peptides recognize molecular markers specifically expressed on tumor tissue, certain peptides, known as tumor-penetrating peptides, initiate a multistep process of extravasation and penetration into the tumor parenchyma.^91^

**Figure 3:**
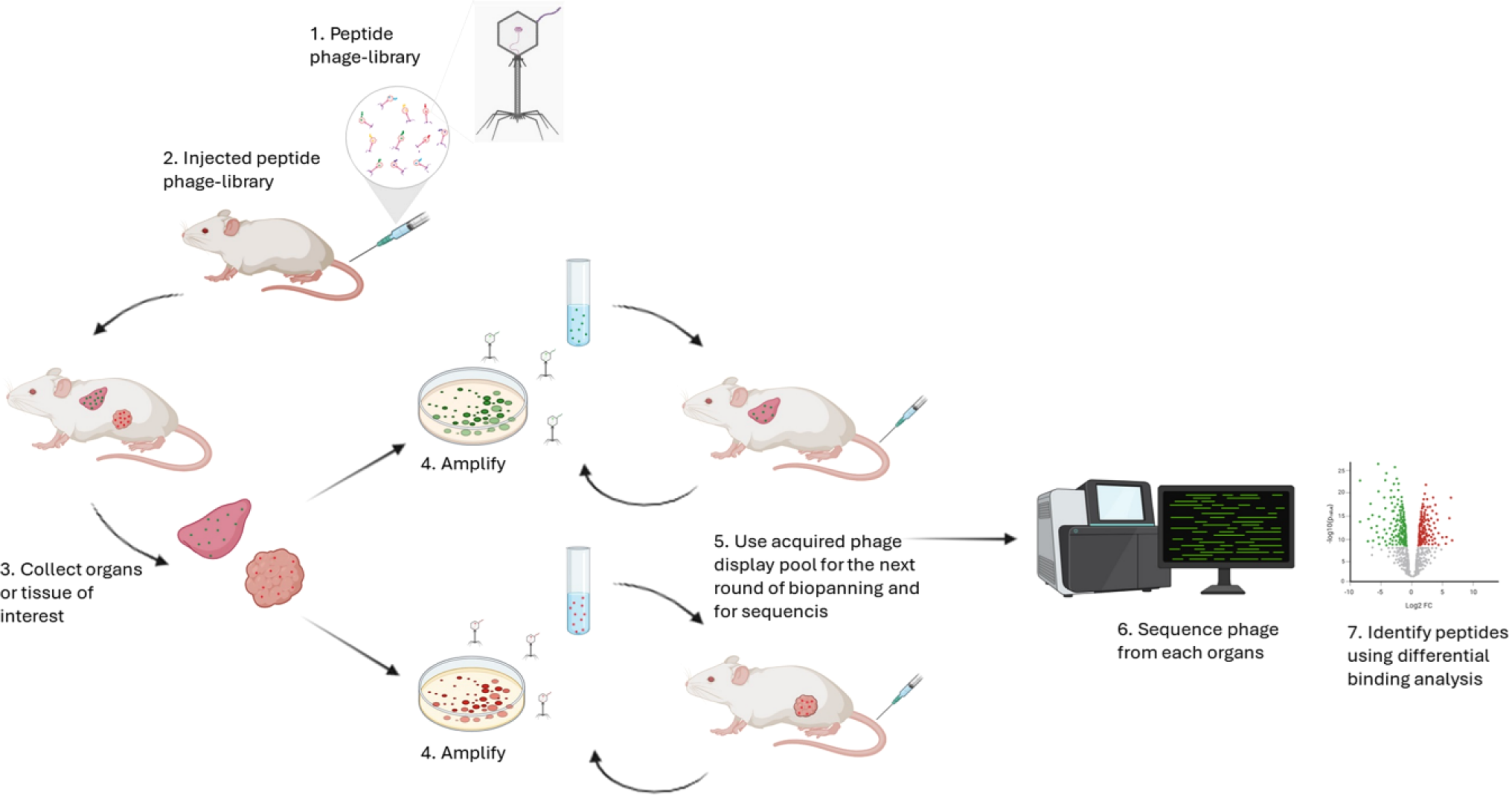
Schematic description of *in vivo* phage display workflow for the identification of tumor-homing peptides. (1) A phage display peptide library is prepared. (2) The library is injected intravenously into the tumor-bearing animals. (3) After circulation, organs or tissues of interest are harvested. (4) Phage particles recovered from each tissue are amplified. (5) The amplified pool is used for subsequent rounds of biopanning and for sequencing. (6) Individual phage clones from each organ are sequenced. (7) Tumor homing peptides are identified through differential binding analysis across tissues. Adapted from.^87,88^ *Created with BioRender.com*.

### 3.1 Tumor Penetrating Peptides: RGD

Among the most prominent examples of tumor-associated systemically accessible targets of affinity ligands are cell adhesion receptors, particularly integrins. These proteins selectively bind to ECM molecules via short peptide motifs, such as the RGD sequence (arginine-glycine-aspartate).^92,93^ Integrins are overexpressed on tumor endothelial cells and can be targeted with RGD and related peptides, enabling the localized accumulation of therapeutic agents in target tissues.^94^

Cilengitide (cyclo-Arg-Gly-Asp-d-Phe-Nal), the first cyclic RGD (cRGD) pentapeptide approved for clinical trials, was evaluated in the Phase III CENTRIC study for newly diagnosed Glioblastoma.^95^ Despite the high therapeutic expectations, it failed to demonstrate a significant improvement in overall survival compared to standard therapy, with a similar therapeutic effect.^96^ This clinical failure demonstrated the limitations of the targeting strategies based solely on receptor binding, thus suggesting that integrin engagement alone is insufficient for achieving meaningful therapeutic outcomes.^97^ Nevertheless, Cilengitide was the proof-of-concept that RGD peptides can be safely administered to patients, reached its integrin targets *in vivo*, and modulated the tumor microenvironment.^98^ Based on this evidence, the research shifted toward tumor-penetrating peptides designed not only to bind specific receptors, but also to promote deep tissue penetration. Among them, iRGD (internalizing RGD) has emerged as a leading compound for the targeted delivery of therapeutics via nanoparticles in solid tumors.^99^

The iRGD peptide was discovered during a search for peptides targeting metastatic prostate cancer using a combined *ex vivo* and *in vivo* phage display approach in nude mice with experimental bone metastases from PC-3 and PPC1 human prostate cancer cells.^100^ After three independent screening rounds, peptides with the sequence CRGDKGPDC were repeatedly found in the enriched pool, indicating their dominance and specific targeting abilities. Further characterization revealed that phage clones expressing a cyclic CRGDKGPDC peptide bound to and internalized into tumor cells, leading to the naming of iRGD, for “internalizing RGD.” The iRGD sequence is unique due to a basic amino acid following the RGD motif (e.g., RGDK/R), creating two overlapping motifs: an integrin-binding RGD and an Neuropilin-1 (NRP-1)-binding C-end Rule (CendR) motif. Mechanistic studies showed that iRGD acts through a three-step process: 1) binding to integrins αvβ3/β5 via the RGD motif, 2) proteolytic processing to expose the C-terminal CendR motif (RGDK/R), and 3) binding of the linearized peptide to NRP-1, enabling transcellular internalization. Each step is crucial for the peptide’s activity, with proteolytic processing reducing the peptide’s affinity for integrins, which allows the CendR motif to bind to NRP-1, triggering the final stage of tumor penetration.^91,101^ The CendR pathway utilized by CendR peptides is similar to macropinocytosis but differs in two keyways: it is receptor-dependent and regulated by nutrient availability via mTOR signaling cascades.^102^ The molecular mechanisms for cell-to-cell transport remain unclear. It has been suggested that CendR payloads might be transported via exosomes or intercellular tube-like structures known as micro/nanotubes, though this remains speculative.^103^

Later, it was found that, similar to iRGD, other peptides also utilize the CendR pathway for tumor homing and penetration. Primary targets for cryptic CendR peptides include αvβ3 and αvβ5 integrins for iRGD, p32/gC1qR for linTT1 and Lyp1, and aminopeptidase N for iNGR. The enzymes responsible for the proteolytic activation of CendR motif, however, remain unknown. In addition, various rational design and screening-based approaches have been used to develop tumor penetrating peptides of novel specificities.^104^ Basically, the emerging theme is that different tumor docking motifs can be combined with cryptic CendR motifs to generate tumor penetrating peptides of novel specificities (Figure 4).

**Figure 4:**
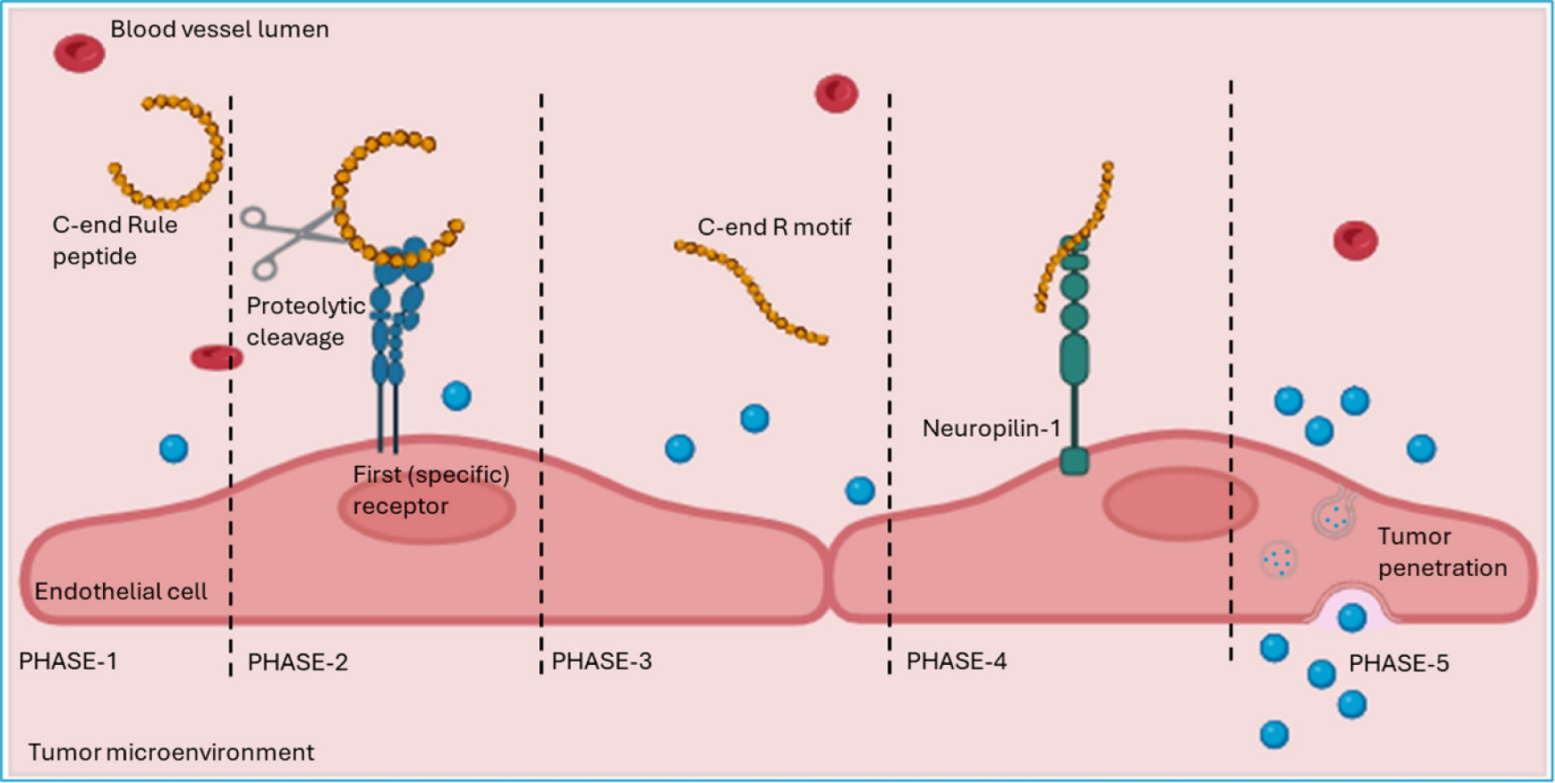
Mechanism of tumor penetration mediated by tumor-penetrating peptides (TPPs), illustrated in five phases (adapted from Figure 1.^105^ Phase 1: TPPs are administered systemically, conjugated to nanoparticles for targeted drug delivery, or coadministered with therapeutic agents. Phase 2: the peptide selectively binds to a primary receptor expressed on the endothelial surface. A tumor-specific protease, such as urokinase (uPA), cleaves the peptide at a specific region. Phase 3: the cryptic CendR motif is exposed. Phase 4: the CendR motif selectively binds to NRP-1, a critical step for receptor-mediated internalization. Phase 5: NRP-1-dependent internalization occurs via a receptor-mediated macropinocytosis-like process that relies on mTOR signaling. Following internalization, the peptide and its therapeutic payload are transported deep into the tumor tissue via transcytosis, enabling effective penetration into the tumor microenvironment. *Created with BioRender.com*.

Multiple studies have demonstrated the therapeutic potential of iRGD-functionalized nanoparticles. Zhang et al., demonstrated that the coadministration of paclitaxel and tetrandrine loaded into hybrid polymer–lipid nanoparticles functionalized with the iRGD peptide overcomes drug resistance in tumor cells, enhancing drug internalization.^106^ Similarly, Hamilton et al. investigated iRGD-targeted NW nanoparticles and found that they significantly impacted the progression of brain metastases and facilitated the retention of nonproliferative tumor cells when administered at early stages of tumor development. These findings suggest a potential application of iRGD-functionalized nanoparticles in the preventive reduction of metastatic spread.^107^ Furthermore, Simon-Gracia et al. demonstrated that iRGD functionalization enhances the efficacy of paclitaxel-loaded polymersomes for the intraperitoneal treatment of peritoneal carcinoma, highlighting its potential for improving drug delivery in challenging tumor environments.^108^

Importantly, there has been rapid progress in translating the prototypic tumor-penetrating peptide iRGD through preclinical studies, all the way to Phase II clinical trials in pancreatic cancer patients and earlier-stage trials in additional solid tumor types.^109^ The iRGD peptide, clinically developed by Lisata Therapeutics in combination with gemcitabine and nab-paclitaxel as Certepetide, has shown promising outcomes in patients with metastatic pancreatic ductal adenocarcinoma. These findings are particularly encouraging when considering the maximum tolerated dose and the associated systemic side effects.^110^ Certepetide has been awarded Fast Track designation (U.S.) and Orphan Drug Designation for pancreatic cancer (U.S. and E.U.), as well as Orphan Drug Designation for glioma, osteosarcoma, and cholangiocarcinoma (U.S.). Additionally, Certepetide has received Rare Pediatric Disease Designation for osteosarcoma (U.S.). These translational efforts have been significantly driven by the ability of iRGD tumor penetrating peptide to potentiate tumor selectivity and efficacy of both conjugated and coadministered therapeutic molecular and nanoscale payloads.^111^ This approach contrasts with most targeting peptides, which require chemical conjugation to nanoparticles to modulate their tropism for precision delivery.

### 3.2 Targeting Cell Surface Proteins with Homing Peptides

Systemic peptide-targetable cell surface receptors are displayed on various cells in tumors, from tumor endothelial cells to malignant and stromal cells. These receptors are often upregulated as part of the angiogenic and proliferative responses and play key roles in tumor neovascularization, growth, and tissue remodeling. Examples include angiogenic integrins, NRP-1, and certain growth factor receptors. Additionally, a common feature in tumors is the aberrant surface expression of intracellular proteins, such as mitochondrial p32 and nucleolar nucleolin, in activated tumor cells.

#### 3.2.1 Integrins

Integrins are a family of transmembrane proteins that function as receptors for ECM components, playing a crucial role in cell-matrix adhesion and intracellular signal transduction. They are heterodimers composed of an α-subunit and a β-subunit, whose combination determines ligand-binding specificity for various ECM proteins, including collagen, FN, laminin, and vitronectin.^112^ In the tumor context, integrins play a crucial role in multiple key processes driving cancer progression, including proliferation, survival, migration, invasion, and angiogenesis. Alterations in integrin expression and activity can promote epithelial-to-mesenchymal transition (EMT), facilitating metastatic dissemination of tumor cells. Additionally, certain integrins, such as αvβ3, αvβ5, and α5β1, are aberrantly expressed in endothelial cells of newly formed blood vessels, contributing to tumor angiogenesis and resistance to antiangiogenic therapies in cancers such as colorectal cancer, nonsmall cell lung carcinoma, glioblastoma, pancreatic cancer, prostate cancer, and melanoma.^98,113^ Many RGD-based amino acid sequences have been developed for the selective targeting of integrins, including the iRGD peptide described above.^114^ For instance, Fang et al., developed doxorubicin-loaded polymeric micelles conjugated with the RGD peptide, demonstrating enhanced targeting and cytotoxicity in osteosarcoma models *in vitro*.^115^ Similarly, Chen et al., reported the use of RGD-modified gold nanoparticles for integrin αvβ3-mediated imaging and therapy of glioblastoma, showing improved accumulation in the tumor sides.^116^ Moreover, recently, Yuan et al., developed polydopamine nanoparticles conjugated with the RGD peptide. *In vivo* studies demonstrate the targeting capability of this nanoparticles, enabling its accumulation at the tumor site. These findings highlight its potential as a promising tool for combined therapy in thyroid cancer.^117^

#### 3.2.2 Neuropilin-1

NRP1 is a transmembrane pleiotropic receptor involved in a multitude of physiological and pathological processes. Under normal conditions, NRP1 is expressed in multiple cell types, including endothelial cells, neurons, and immune cells. It plays a crucial role in nervous system and vascular development by acting as a coreceptor for growth factors such as vascular endothelial growth factor (VEGF) and semaphorins, thereby regulating angiogenesis and axonal guidance. Additionally, NRP1 is implicated in immune response modulation and vascular permeability regulation.^118^ NRP1 is overexpressed in various cell types across solid tumors, including glioblastoma, prostate, lung, breast, and pancreatic cancers. Its interaction with VEGF and other ligands promotes tumor angiogenesis, fostering a microenvironment that supports tumor growth. Furthermore, NRP1 contributes to tumor cell proliferation, migration, and invasion, thereby enhancing metastatic progression. Due to its ability to interact with multiple receptors and signaling pathways, NRP1 has emerged as a promising therapeutic target for strategies aimed at inhibiting angiogenesis and tumor dissemination.^119,120^

In the context of affinity targeting, NRP-1 was first identified as the receptor for the prototypic CendR peptide, RPARPAR. Its relevance to the activity of tumor-penetrating peptides was later demonstrated. These peptides contain a cryptic C-end Rule (CendR) motif (R/KXXR/K) that, upon proteolytic activation, acquires the ability to engage the b1b2 domain of NRP-1, enhancing vascular permeability and trans-tissue transport. As discussed above, targeting NRP-1 via the CendR pathway has been shown to improve the delivery of therapeutic payloads, including small molecules, imaging agents, and nanoparticles, facilitating deeper tumor penetration.^100,121^

*In vitro* tests have demonstrated that doxorubicin-loaded niosomes functionalized with an RPAR (sequence: RPARPAR) a peptide that directly binds neuropilin-1, selectively bound to cells positive for this receptor and exhibited enhanced cytotoxic activity in these receptor-positive cells.^122^

#### 3.2.3 p32 (gC1qR/HABP)

p32 is a 32 kDa trimeric chaperone that plays a crucial role in cellular metabolism and signaling. Within mitochondria, p32 shifts metabolism from oxidative phosphorylation to glycolysis and regulates kinase activity.^123^ It is a multifunctional, multicompartmental protein, while predominantly localized in mitochondria of quiescent cells, and it is markedly overexpressed on the surface of tumor cells and endothelial cells, both in the blood and lymphatic vasculature. High p32 expression has been reported in various malignancies, including ovarian and breast carcinomas, endometrial cancer, melanoma, prostate cancer, and glioblastoma. Its distinct expression pattern in cancer and its role in tumor metabolism make p32 a promising target for therapeutic and diagnostic applications.^104^ In the context of affinity targeting, p32 was first identified as the receptor for the LyP-1 peptide (CGNKRTRGC), which selectively targets malignant cells, tumor-associated lymphatics, and macrophages.^124^ Notably, Song et al. developed Lyp-1-modified dendrimers labeled with ^1 3 1^ I, demonstrating their potential as a theranostic platform for cancer diagnosis and treatment.^125^ Another peptide, CgKRK, has also been shown to bind p32 selectively. In particular, Mashreghi et al. enhanced the delivery of PEGylated doxorubicin by leveraging dual targeting of both tumor and tumor-associated endothelial cells through an anti-p32 peptide. *In vivo* studies showed an increased drug accumulation of these nanoparticles compared to Caelyx, thus leading to improved survival rates and reduced systemic side effects.^126^ More recently, the development of the p32-targeting TT1 peptide and its linear form, LinTT1, (sequence: AKRGARSTA) has further expanded the potential of p32-targeting strategies. These peptides also feature a cryptic internal RGAR motif that is C-terminally exposed upon proteolytic cleavage by urokinase-type plasminogen activator (uPA), to trigger neuropilin-1 (NRP1). This mechanism facilitates parenchymal diffusion and cellular internalization of the peptide. Säälik et al. demonstrated the effectiveness of p32 targeting in glioblastoma models by functionalizing TT1 with silver nanoparticles and albumin-paclitaxel nanoparticles, resulting in enhanced tumor accumulation and reduced tumor activity.^127^

Nanoparticles conjugated with LinTT1 have been extensively investigated in preclinical studies for selective targeting of breast cancer.^128,129^ These studies have demonstrated an increased selectivity and cellular penetration of nanoparticles both *in vitro* and *in vivo*, endorsing their effectiveness in anticancer therapy. LinTT1 has also been functionalized on NW, proving to be a promising candidate for potential glioblastoma therapy.

LinTT1 has also been functionalized on NW for the selective treatment of glioblastoma, showing encouraging *in vivo* results and emerging as a promising strategy for GBM therapy.^130^

### 3.3 Emerging Theme: The Tumor Extracellular Matrix as a Target for Affinity Ligands

The ECM represents an abundant target for various affinity ligands, including peptides. As a complex and dynamic network, the ECM plays a fundamental role in cell growth, differentiation, migration, and is deeply involved in pathological processes such as tumor invasion and metastasis.^131^ Structurally, the ECM is composed of a diverse array of fibrous proteins, glycosaminoglycans (GAGs), and proteoglycans, and a multitude of their splice variants^132^ some of which serve as targets for tumor-penetrating peptides.^105^

#### 3.3.1 Fibronectin

Fibronectin (FN) is an abundant, multifunctional glycoprotein within the ECM, existing primarily as a 250–280 kDa dimer. It plays critical roles in cell adhesion, ECM organization, wound healing, and angiogenesis.^133^ Importantly, FN has also been implicated in the survival, proliferation, and invasion of malignant cells, making it a promising target for tumor-specific therapies.^134^ Structurally, FN is a modular protein composed of three repeated domains, termed type I, type II, and type III repeats, each harboring binding sites for ECM components and cell surface receptors.^135^ Alternative splicing of FN pre-mRNA generates up to 20 distinct isoforms, particularly during embryogenesis and tumorigenesis. Among these, the oncofetal FN isoforms, specifically the extra domain A (EDA-FN) and extra domain B (EDB-FN), have gained considerable attention due to their tumor-restricted expression. Unlike in normal adult tissues, these isoforms are highly expressed in most solid tumors and in angiogenic vasculature, making them attractive biomarkers and therapeutic targets.^136^ A promising EDB-FN ligand is ZD2, a small peptide identified through phage display screening. ZD2 exhibits high specificity for EDB-FN and has been developed for prostate cancer targeting. A ZD2-Cy5 conjugate has been synthesized for molecular imaging of prostate cancer, demonstrating selective and efficient tumor binding both *in vitro* and *in vivo*.^137^

#### 3.3.2 Tenascin-C

Tenascin-C (TNC) is an extracellular matrix (ECM) glycoprotein with distinct roles in both physiological and pathological conditions. Under normal conditions, TNC is highly expressed during embryonic development, where it regulates cell migration and tissue organization. In adult tissues, its expression is either absent or minimal, but it is significantly upregulated in response to tissue injury, contributing to inflammatory and regenerative processes.^138^ In contrast, TNC plays a crucial role in tumorigenesis. It interacts with cell surface receptors and ECM components, influencing cell adhesion, migration, and invasion. Furthermore, TNC is actively involved in tumor angiogenesis, promoting the formation of new blood vessels that sustain tumor growth and metastasis. Additionally, TNC contributes to tumor microenvironment remodeling, creating conditions that enhance cancer cell survival and proliferation. Notably, TNC upregulation correlates with higher tumor grades and is associated with poor prognosis in multiple malignancies.^139^ TNC is overexpressed in high-grade brain tumors, including astrocytoma and glioblastoma, as well as in various carcinomas such as lung cancer, renal carcinoma, malignant melanoma, and urothelial carcinoma. Due to its tumor-restricted expression, TNC has been extensively explored as a target for selective drug delivery.^140^

Several TNC-targeting molecules have been developed, including single-chain variable fragment (scFv) antibodies and tumor-homing peptides. Lingasamy et al. developed a dual-targeting peptide, PL1 (sequence: PPRRGLIKLKTS) capable of interacting selectively with both fibronectin extra domain B (FN-EDB) and TNC-C. This dual specificity enables robust and precise delivery of both imaging agents and therapeutic payloads to solid tumors overexpressing these ECM markers. Specifically, iron oxide nanoworms (NW) conjugated with PL1 were tested in *in vivo* models of glioblastoma and prostate cancer, demonstrating selective tumor accumulation. When functionalized with the pro-apoptotic KLAKLAK peptide, these NW-based nanocarriers exhibited potential antitumor activity.^141^

More recently, PL3 (amino acid sequence: AGRGRLVR) has been developed and analyzed for its selective interaction with TNC. Interestingly, PL3 contains a C-terminal RXXR motif, known to target the neuropilin-1 (NRP1) receptor, which is implicated in cell and tissue penetration. PL3 was conjugated to nanoworms (NW) and tested in xenograft models of prostate cancer and gliomas, both of which overexpress TNC and NRP1. Experimental analyses demonstrated highly selective and efficient tumor accumulation of functionalized nanoparticles, as well as potential antitumor effects when conjugated with pro-apoptotic peptides.^142^ Notably, Tobi et al., developed derivatives of the PL3 peptide that bind to NRP1 following proteolytic cleavage by uPa while retaining affinity for the TNC receptor. *In vitro* binding and internalization assays, along with *in vivo* biodistribution studies in orthotopic glioblastoma-bearing mice, confirmed the efficacy of the novel peptides PL3uCendR and SKLG, demonstrating uPa-dependent binding to NRP1 and reducing off-target interactions in healthy tissues compared to PL3.^143^

#### 3.3.3 Hyaluronic Acid

Hyaluronic acid (HA) is a nonsulfated glycosaminoglycan and a fundamental component of the ECM, present in various tissues. It is a high-molecular-weight molecule, approximately 106 kDa, synthesized by the integral membrane glycosyltransferase enzyme hyaluronan synthase and degraded by hyaluronidase enzymes. Under physiological conditions, HA contributes to tissue hydration and elasticity, facilitating cell migration and proliferation.^144^ In oncology, HA plays a significant role in tumor progression by influencing cell adhesion, migration, and invasion.^145^ It interacts with receptors overexpressed on tumor cells, such as CD44, a transmembrane glycoprotein that serves as the principal cell surface receptor for HA, and the receptor for hyaluronan-mediated motility (RHAMM), also known as CD168, regulates several cellular processes, including cell motility, proliferation, and the organization of the mitotic spindle.^146^ Given these interactions, HA-based systems have been explored as potential strategies for targeted drug delivery in cancer therapy. By conjugating therapeutic agents to HA, it is possible to exploit the affinity between HA and its receptors, facilitating the selective delivery of drugs to tumor cells. Additionally, the enzymatic degradation of HA within the tumor microenvironment can provide a controlled release mechanism for the therapeutic payload, enhancing the efficacy and specificity of the treatment. Peritoneal carcinomatosis is a condition that occurs because of local dissemination and subsequent spread of epithelial cells derived from gastric, colon, ovarian, and pancreatic carcinomas within the peritoneal cavity. The IP3 peptide (sequence: CKRDLSRRC), which binds to HA, has been functionalized on silver nanoparticles, enabling targeted delivery to peritoneal tumors of gastric and colon origin. These findings suggest potential applications for the selective delivery of therapeutics to peritoneal malignancies.^147^

All the peptides discussed are listed in Table 3, along with their amino acid sequences, structures and targets.

**Table 3:** Peptide Structure Including Sequence, Structural Characteristics, and Molecular Target

Peptide Name	Sequence	Type	Molecular Target
RGD	Arg-Gly-Asp	Linear	Integrins αvβ3, αvβ5
cRGD	Cyclo(RGDfK)	Cyclic	Integrins αvβ3, αvβ5
iRGD	CRGDKGPDC	Cyclic	Integrins + Neuropilin-1
LyP-1	CGNKRTRGC	Cyclic	p32/gC1qR
TT1	CKRGARSTC	Cyclic	p32/gC1qR
LinTT1	AKRGARSTA	Linear	p32/gC1qR
RPARPAR	RPARPAR	Linear	Neuropilin-1
PL1	CRKRLDRNC	Cyclic	FN-EDB and TNC-C
PL3	CGRLYGPC	Cyclic	TNC
IP3	CPATSFQDC	Cyclic	HA
NGR	Asn-Gly-Arg	Linear	CD13/aminopeptidase N
ZD2	–	Cyclic	EDB-fibronectin extracellular matrix

## 4 Preclinical Validation of Peptide- Conjugated Nanocarriers

Recent *in vivo* studies have consistently demonstrated that decorating nanoparticles with tumor-homing peptides significantly increased the antitumor efficacy compared to free drugs. For instance, C-peptide-decorated solid lipid nanoparticles loaded with paclitaxel decreased of 82% the breast tumor volume in 4T1 engrafted murine tumor models compared to the 36% of reduction obtained with free paclitaxel, and minimizing the systemic toxicity.^148^ Similarly, iRGD-functionalized silica/gold nanoparticles, loading paclitaxel, had a prolonged tumor retention and significantly increased the tumor growth inhibition in murine breast cancer models.^149^ In another example, SMAC peptide-doxorubicin prodrug-encapsulated inside liposomes (Aposomes) caused a complete regression of colon cancer xenografts, in contrast to the moderate volume decrease obtained with free doxorubicin.^150^ A6 peptide-conjugated PLGA–PEG nanoparticles, codelivering doxorubicin and anti-miR-21, increased the drug intratumoral accumulation and the substantial tumor shrinkage in triple-negative breast cancer models.^151^ Short antiangiogenic MMP-2 peptide-functionalized superparamagnetic iron oxide nanoparticles (SPIONs) loaded with paclitaxel yielded a 69.7% tumor inhibition rate in A549 xenograft mice compared to Taxol and prolong *in vivo* the drug’s half-life.^152^ Likewise, dual AS1411 aptamer/RGD-functionalized chitosan-PLGA nanoparticles coloaded with docetaxel and up conversion nanoparticles lack the growing of brain tumor in BALB/c nude mice with minimal systemic toxicity.^153^ Additionally, polymeric nanocarriers conjugated with dual cell-penetrating peptides delivered miR-205–5p in the cutaneous squamous cell carcinoma xenografts of murine model and caused a significant tumor regression with minimal off-target effects compared to free miRNA.^154^

All these results demonstrated the therapeutic potential of peptide-mediated nanoparticle targeting to improve drug delivery specificity, intratumoral accumulation, and overall antitumor efficacy *in vivo*.

## 5 Concluding Remarks: From Innovation to Clinical Translation in Nanomedicine

Nanomedicine has progressed from the early vision of “magic bullets” to clinically approved platforms, such as liposomal doxorubicin, albumin-bound paclitaxel, and polymeric micelles, that increase the drug solubility, circulation half-life, and therapeutic index through controlled release of payloads.^155^ The development of antibody-drug conjugates and active targeting drug delivery systems has refined the biological “lock-and-key” paradigm, where the molecular receptors on tumor cells act as locks, while targeted agents, from antibodies to peptides, serve as keys granting selective access to malignant tissues.^156^

Over the past decade, tumor-homing peptides (e.g., iRGD, NGR, LyP1) identified using phage display have emerged as specific ligands comparable to monoclonal antibodies but with simpler synthesis and lower manufacturing costs. Preclinical studies demonstrate that decorating nanocarriers with these peptides can boost tumor accumulation 3–5 fold relative to untargeted nanocarriers, thus highlighting their capability to overcome biological barriers, heterogeneous uptake and off-target toxicity.^157^

Translating these advances from bench to bedside remains a formidable challenge. The tumor microenvironment, with its abnormal vasculature, dense stroma, and immunosuppressive niches, impedes nanoparticle penetration. Moreover, immune recognition, peptide stability, and the need for largescale, good manufacturing practice-compliant manufacturing (including microfluidic synthesis) demand further optimization. Nanocarrier platforms with homing peptides offers a promising path forward. By combining the tissue specificity of peptides, the “keys”, with the controlled release and payload versatility of nanoparticles, the “vehicles”, this integrated approach can address the limitations of each modality, reducing offtarget toxicity and improving manufacturing consistency. This synergy will require rigorous optimization of conjugation chemistries, nanoparticle physicochemical parameters (size, charge, surface density), and scalable production methods.

As the field advances, deepening our understanding of biological interfaces and manufacturing technologies will be essential. Only by confronting these technical and translational hurdles can the full promise of precision nanomedicine be realized, paving the way for next-generation cancer therapies with maximized efficacy and minimized toxicity.
